# Partial Least Squares Regression-Based Robust Forward Control of the Tableting Process

**DOI:** 10.3390/pharmaceutics12010085

**Published:** 2020-01-20

**Authors:** Yusuke Hattori, Miki Naganuma, Makoto Otsuka

**Affiliations:** Research Institute of Pharmaceutical Sciences, Faculty of Pharmacy, Musashino University, 1-20 Shin-machi, Nishi-Tokyo city, Tokyo 202-8585, Japan; s1443033@stu.musashino-u.ac.jp (M.N.); motsuka@musashino-u.ac.jp (M.O.)

**Keywords:** feed-forward control, near-infrared spectroscopy, partial least squares regression, tableting, high-shear wet granulation, robust control, process data analysis

## Abstract

In this study, we established a robust feed-forward control model for the tableting process by partial least squares regression using the near-infrared (NIR) spectra and physical attributes of the granules to be compressed. The NIR spectra of granules are rich in information about chemical attributes, such as the compositions of any ingredients and moisture content. Polymorphism and pseudo-polymorphism can also be quantitatively evaluated by NIR spectra. We used the particle size distribution, flowability, and loose and tapped density as the physical attributes of the granules. The tableting process was controlled by the lower punch fill depth and the minimum distance between the upper and lower punches at compression, which were specifically related to the tablet weight and thickness, respectively. The feed-forward control of the process would be expected to provide some advantages for automated and semi-automated continuous pharmaceutical manufacturing. As a result, our model, using a combination of NIR spectra and the physical attributes of granules to control the distance between punches, resulted in respectable agreement between the predicted process parameters and actual settings to produce tablets of the desired thickness.

## 1. Introduction

In the field of pharmaceutical technology, continuous manufacturing from powder blending to the compression process is anticipated as the next-generation of solid dosage production [1,2]. Continuous manufacturing is a new and increasingly first-choice option to produce pharmaceuticals. The merits of continuous manufacturing are the flexibility in the production volume and the quality guarantee based on the monitoring data. The volume can be controlled by the rate and time of production.

Process analytical technology (PAT) is integral to the system of continuous manufacturing. The PAT framework was introduced by the FDA in 2004 [3]. Subsequently, the concepts of real-time release testing (RTRT) and quality by design (QbD) were introduced in the ICH Guidelines ICH Q8(R2), and the use of these concepts was recommended by the FDA in 2009 [4]. The essential feature of PAT, RTRT, and QbD is to adjust the quality of the final products by process design and control. The FDA strongly recommended that process control be conducted using real-time process monitoring with confirmed scientific approaches.

Near-infrared (NIR) spectroscopy is one of the most confirmed and prevailing technologies for the real-time monitoring of specific aspects of product quality, such as moisture content [5,6] and blend uniformity [7,8,9,10]. The chemical attributes of solid formulations are precisely represented in the NIR spectra. Moreover, crystallographic information of solids, such as the presence of polymorphism [11,12,13] or pseudo-polymorphism [14,15,16], can be revealed by NIR spectroscopy. 

Real-time monitoring data is valuable not only to ensure product quality, but the monitoring data is also useful for advanced process controls such as RTRT and the feed-forward control [17,18,19,20]. Advanced or active process control is especially useful and compatible with continuous manufacturing. Because continuous manufacturing operations are either automated or semi-automated, it is preferable that the process parameters are adjustable in response to either expected or unexpected disturbances. For example, the moisture content and particle size of materials strongly affect their flowability, and the process of wet granulation and subsequent drying must be seriously controlled depending on the moisture content, particle size, and density of the granules. Moreover, the polymorphisms of active pharmaceutical ingredients (API) and additives may contribute to the pharmaceutical properties of granules and tablets, such as their compactibility [21,22,23] and the bioavailability of API [24,25]. The variation in the contents of all ingredients is also a critical quality factor affecting the process control. As mentioned above, NIR spectra can be used to predict these variations in the chemical attributes of materials. 

Researchers typically use NIR spectra to determine one or more of the characteristics of materials with multivariable analysis such as the partial least squares regression (PLSR) method [26]. The moisture content, polymorphism, and contents of all the ingredients are predictable by the NIR spectra of materials and PLSR; however, these predicted values are only reliable within a margin of error to ensure the quality of the products. For the active control of the manufacturing process, it is not efficient to use the predicted values, because the prediction is sure to include a certain level of error in the value. The prediction of control parameters using the predicted value over and over again results an amplification of the prediction error and reduction in accuracy. Thus, it is rationalized with less error for active control to use NIR spectra without any attribute prediction. In this study, we demonstrate how to utilize NIR for the monitoring of information for the robust feed-forward control of tablet compression. 

Usually, tablet compression is controlled by adjusting the moving distance of the upper and lower punches. The lower punch fill depth (FD) controls the loading amounts of granules in the die, which are used to determine the tablet weight and the content of API. The compression pressure is controlled by the minimum distance between the lower and upper punches (MDPs) at compression. Thus, the important point is how to determine the moving distance of the punches based on the physical and chemical attributes of the granules. 

In our previous work, we tried to calibrate the compression process parameters for extruded granules by the PLSR method using the NIR spectra and physical attributes of the granules [20]. We successfully provided the design spaces of the FD and MDPs for the desired weight and thickness of the tablets. However, the precision and accuracy of the parameter prediction by the PLSR calibration model were low, and the model showed a low level of robustness for the application even for internal validation. It was considered that the low precision and accuracy were caused by the small variations in the sample formulation and characteristics of the training set. 

In this study, we establish a robust calibration model for controlling the tableting process parameters in order to produce optimal tablets of the desired weight and thickness by PLSR. The granules are prepared by high-shear wet granulation with large variations in the granulation conditions of material formulation, rotational speed of side-screw, additive amount of water, and drying time of wet granules. The high-shear wet granulation is more suitable and widely used to prepare granules for compression. The NIR spectra of the granules are often used to determine the chemical attributes. Here, because we do not aim to guarantee the product quality, the NIR spectra are used without quantifying the attributes. Additionally, the physical attributes of particle size distribution, loose bulk density *ρ*_L_, tapped density *ρ*_T_, and flowability of the granules are used as the variables for the process parameter regression. Finally, we try external validations of predicting the optimum process parameters of compression by the PLSR-based forward control. 

## 2. Materials and Methods 

High-shear wet granulation was performed to prepare the granules with anhydrous lactose (AL), corn starch (CS), hydroxypropyl cellulose (HPC), micro-crystalline cellulose (MCC), and loratadine (LTD). AL (21-AN) and HPC (L grade) were kindly provided by DFE Pharma (Goch, Germany) and Nippon Soda (Tokyo, Japan), respectively. CS and MCC (PH-102) were purchased from Japan Corn Starch (Tokyo, Japan) and Asahi-Kasei Chemicals (Tokyo, Japan), respectively. LTD was kindly provided by Ohara Pharmaceutical (Shiga, Japan). Magnesium stearate (MgSt) was purchased from FUJIFILM Wako Pure Chemicals (Osaka, Japan).

Three formulations (I, II, and III) were prepared as shown in Table 1 and used for granulation without MgSt. The blending process was performed using a high-shear wet granulator at an agitation speed of 200 rpm for 5 min. Three hundred grams of Form I powder blend was used for granulation, which was performed using the 9 different combinations of process parameters listed in Table 2. In the granulation process, 10 mL of water was added 7 or 8 times per 1 min with agitation at 200 rpm, and these numbers were fixed for all the formulations and test runs. We varied the speed of the side cross-screw, as shown in Table 2, in order to control the particle size and density of the granules. The wet granules were dried for 2 or 5 h in an oven at 70 °C.

The granulation and drying of both Forms II and III were performed using two different combinations of process parameters, as shown in Table 2. In total, 13 batches of granules, including 9, 2, and 2 batches of Forms I, II, and III, respectively, were obtained. Prior to the tableting of granules, 1 wt % of MgSt was added to the granules and blended for 3 min. Approximately 5 g of the mixture was sampled and put on a glass dish to measure NIR spectra. The NIR spectra of the mixture were collected from the bottom of the sample dish using an FT–NIR spectrometer (MPA; Bruker Optics, Ettlingen, Germany) with the diffuse-reflectance method. The spectral collection was performed between 4000 and 9500 cm^−1^ with the resolution of 16 cm^−1^. For each sample, 6 spectra were collected with changing the collecting position and averaged.

The mass standard size distributions of granules were measured by the sieving method. The median size (D50) and the diameters at 10% (D10) and 90% (D90) of the cumulative distribution were determined. The loose and tapped density of the granules were measured using a 50-mL-cylinder. The loose bulk density was obtained as the ratio of the mass of the granules divided by the gently funneled volume in the cylinder. The granule-funneled-cylinder was tapped 70 times from a height of approximately 1 cm. The tapped density was the density after the tapping. The flowability of the granules was evaluated by the Hausner ratio and Carr index [27]. 

The tableting process was performed by varying the tableting parameters of displacement of the lower and upper punches using a single punch tableting machine (TAB ALL; Okada Seiko, Tokyo). The inner diameter of the die was 8 mm, and flat-faced punches were used for compression. The lower punch fill depth and upper punch displacement were adjusted to obtain the desired weight of the tablet and to apply the desired compression pressure, as shown in Table 3, respectively. Both parameters of the lower and upper punches were recorded as $y_{L}$ and $y_{U}$, respectively. The distance between the punches at compression was given as the difference between the upper and lower punches as $∆y=y_{L}-y_{U}$. As listed in Table 3, six different combinations of tablet weight and compression pressure were used for each batch of granule. Finally, 54, 12, and 12 batches of tablets were obtained for Forms I, II, and III granules, respectively, and for each batch, 30 tablets were prepared and used to determine tablet thickness and weight. 

PLSR-based process control models were prepared with the independent variables of NIR spectral data and/or physical attributes of granules as the input variables using the Unscrambler X (Camo Analytics, Oslo, Norway). In principle, the multivariable analysis was represented as follows [20]:

$$\left(\begin{array}{c} y_{1}\\ \vdots \\ y_{n} \end{array}\right)=\left(\begin{array}{ccc} x_{11} & \cdots & x_{1i}\\ \vdots & \ddots & \vdots \\ x_{n1} & \cdots & x_{ni} \end{array}\;\begin{array}{ccc} x_{1i+1} & \cdots & x_{1i+j}\\ \vdots & \ddots & \vdots \\ x_{ni+1} & \cdots & x_{ni+j} \end{array}\;\right)B_{PLS},$$

where *x*, *y*, and *B_PLS_* indicate the independent variables, process parameters, and a regression coefficient obtained by PLSR, respectively. The subscript *n* is the number of samples. The subscripts *i* and *j* are the number of input and output variables, respectively. As the output variables, the weight and thickness of each tablet were measured and used for the calibration models, and the desired weight and thickness were used for the process parameter prediction. 

The sets of FD $y_{L}$ and MDPs ∆y were entered into the process parameter data set as the dependent variables. For the preparation of the calibration model, the training set included 54 batches of tablet using 9 granule batches listed in Table 2, and 30 tablets of each batch were entered into the data set. Moreover, another calibration model using 48 batches of the tablet was prepared excluding the batches of Form II (Run No. 7) to perform external validation using the data set of Form II batches (Run No. 9). In total, the training data set was composed of 1620 and 1440 samples including and excluding Form II batches, respectively. The test set for prediction included 24 batches of tablet including 4 granule batches of Run No. 1, 5, 8, and 9. All the data was processed with mean centering prior to the PLSR calculation, and the validation was performed using the random-cross-validation method with 20 segments.

## 3. Results and Discussion

The physical attributes of the prepared granules were measured and listed in Table 4. The effect of the rotation speed of the side cross-screw on the physical attributes was not very important; however, the volume of water addition impacted the particle size distribution. It seems that the particle size becomes larger with a larger volume of water addition. For example, the granules of Run No. 2, 3, and 6 with 80 mL of water addition resulted in a larger size compared to the granules of Run No. 1, 4, and 4 with 70 mL of water addition. This result is consistent with our previous report [27]. The loose bulk density was varied by controlling the drying time, and long-time drying resulted in low-density granules. By controlling the process parameters of high-shear wet granulation, the granules were obtained with a wide batch of physical attributes.

The calibration models of FD and MDPs were prepared by PLSR using NIR spectral data and/or the physical attributes. The calibration and validation results are summarized in Table 5. For the calibration models of FD and MDPs, 6 and 5 latent variables (LVs) were used respectively to obtain a linear relation between the prediction and reference variables with less root mean squared error (RMSE). In this study, it is difficult to determine the adequate number of LVs for calibration because the dependent variable is the process parameters for which the contributors are not specified, unlike with predicting concentration. Therefore, the number of LVs was determined to be the lowest number providing a flat determination coefficient within 1% of the variation when the coefficients were plotted as a function of the number of LVs. Additionally, the noise in the loading weight and regression vectors informs us of the result overfitting; thus, the loading weight vectors of the NIR spectra were concerned to involve less noise [28].

In the comparison among the types of independent variable data sets, the NIR spectra data set of chemical attributes and combination of physical and chemical attributes models represented a similar and lowest RMSE for the calibration and validation of both FD and MDPs. When the data set of Form II was excluded to prepare the calibration models, the quality of the models were almost comparable with the model including the data set.

In our previous study [20], the standard errors of validation using different combinations of input attributes were 0.368 mm and 0.121 mm for predicting optimum FD and MDPs, respectively. Comparing these results with our present findings using the combination model, the current validation lowered the error to approximately one-third of the previous error for both the combination models. Moreover, the coefficient of determination *R*^2^ was considerably improved to greater than 0.969. The use of a wider range of formulations may have contributed to a higher-precision model.

**Figure 1:**
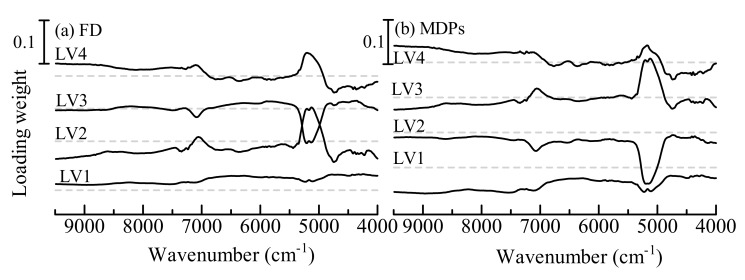
The loading weight of the first four latent variables (LVs) in near-infrared spectra for the control model of fill depth (FD) (**a**) and minimum distance between punches (MDPs) (**b**). The gray dashed lines indicate the zero-weight level for each LV.

The loading weight vectors in the NIR spectra of the first 4 LVs are shown in Figure 1. The first LV loading weight vectors of FD and MDPs indicated broad peaks over the spectral range 4000–9500 cm^−1^. The broad weight means that the baseline shift of spectra contributes to the calibration model. The baseline shift is mainly caused by changes in the size and density of the granules. The peaks of other loading weights were due to the OH of water around 5200 cm^−1^ and 7100 cm^−1^, which represents the contribution of the change in moisture content. The second LV of the FD model and third LV of the MDPs model denoted the peak due to the OH of water at 4800 cm^−1^, which corresponds to water molecules with a strong interaction of hydrogen bonding. Hence, these LVs indicate the contributions of not only the moisture content but also the deviations in the composition of hydrated ingredients such as starch and lactose. The fourth LV of both models included small peaks around 6500 cm^−1^, which are attributed to the contributions of the CH stretching mode of ingredients. As well as the contributions of hydration, the deviations in the alkyl of ingredients contribute to the models.

**Figure 2:**
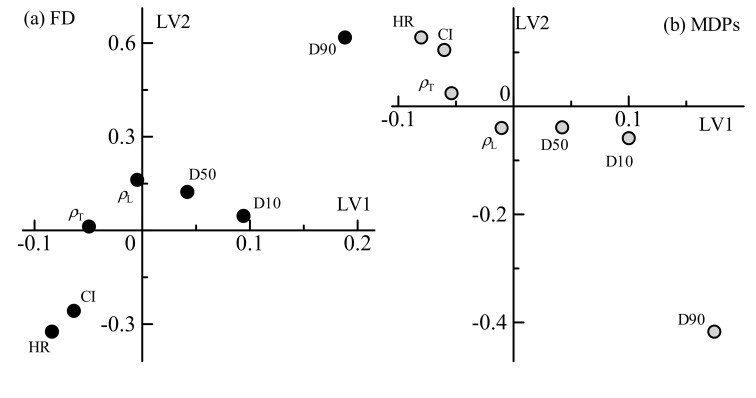
Loading plots of the first and second LVs in the physical attributes of granules for the control model of FD (**a**) and MDPs (**b**). HR and CI indicate the Hausner ratio and Carr index, respectively.

Figure 2 shows loading plots of the first and second LVs of the physical attributes. In the scatter plots of loading weight, the distance from the zero level is correlated with the rate of contribution to modeling. The loadings of FD and MDPs denoted similar patterns. Both the Hausner ratio and Carr index represent the degree of powder flowability, with larger numbers of either representing lower flowability. Both the first and second LVs of the FD model denoted negative contributions of flowability. Every other variable of density and particle size distribution also contributed to the models. Loose bulk density, tapped density, and D50 were weakly related. The values of D10 and D90 were related to both models, and these contributions were contrasted with flowability. The flowability was dependent on the particle size and moisture content; thus, it is reasonable to relate the Hausner ratio and Carr index with these variables and the loading weights of moisture content in the NIR spectra. On the other hand, the Hausner ratio and Carr index denoted higher contributions to the control models than the density. Even though these values are calculated from the density values, the ratio between loose and tapped densities may be effectual for the modeling.

Since the FD is a reliable index of a tablet weight, the high contribution of density is an inevitable result. However, it may be necessary to reconsider the contributions when using a rotary tableting machine or applying precompression. Precompression removes air from the powder bed and leads to a rearrangement of particles.

The process parameters were predicted for the desired values of tablet weight and thickness, which were entered as independent variables of the prediction data set. The tablet weight values were desired as 180, 200, and 220 mg, and the thickness values were given as between 2.6 mm and 3.6 mm depending on the tablet weight. The prediction errors were evaluated by comparing them to the actual values and shown in Table 6. The slope and offset were calculated as the relation between the actual and predicted values, and the combination model provided the best slope close to 1 and the smallest offset among the models for FD. The RMSE of prediction and bias were also minimized by the combination model. Despite the low RMSE of calibration and validation, the NIR model resulted in a larger deviation than the combination model. The contribution rate of the physical attributes for the FD prediction may be higher than that of the chemical attributes. The NIR spectra can involve information related to the particle size and density as shift in the baseline; however, it is suggested that the baseline shift may be insufficiently robust to predict FD. For the prediction of MDPs, these values by three models were in the comparable level, and the MDPs prediction resulted in smaller errors than the FD prediction. Although external validation using the combination model (-) resulted in a lower quality of prediction than the internal validation of other models, the internal validation of the combination model (+) denoted a lower RMSE and bias than the other models for both FD and MDPs.

**Figure 3:**
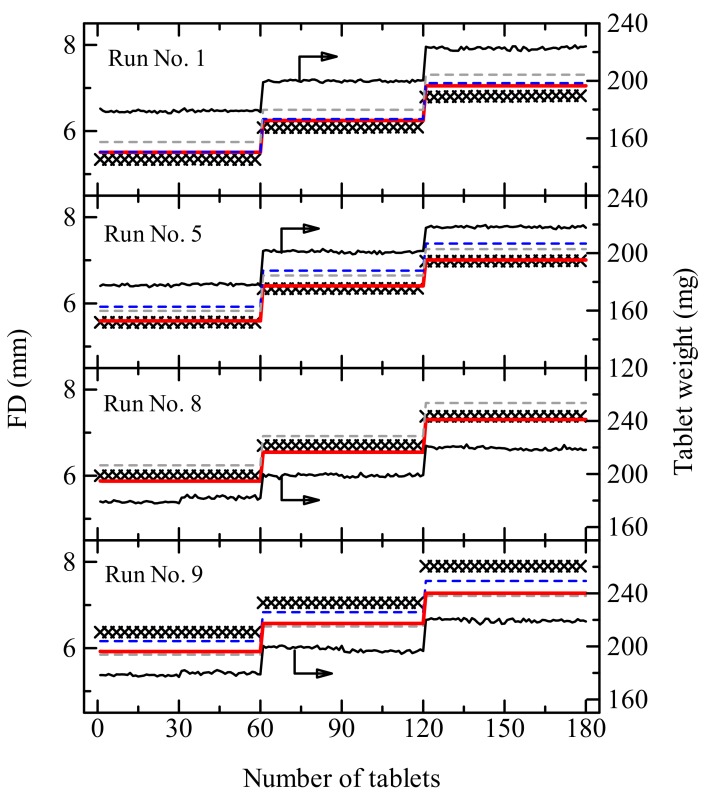
Actual setting (×) and predicted values of FD for the desired tablet weights of 180, 200, and 220 mg by the NIR model (gray dashed line), physical attribute model (blue dashed line), and combination model (red solid line). The solid back line indicates the actual weight of tablets. The prediction was performed for batches of the four runs (No. 1, 5, 8, and 9) shown in Table 2. The prediction of No.9 data was performed as an external validation using the calibration model prepared excluding run No. 7 data.

Figure 3 shows the results of the FD control for a desired weight of tablet. The predicted results derived using the combination model showed a respectable agreement with the actual settings. On the other hand, the Form II excluded model resulted in a lower accuracy of FD prediction. The external validation denoted unsatisfied results; however, the FD values were finely predicted by the Form II included model. Hence, it is strongly required to assemble a huge number of data sets for the robust prediction of FD.

**Figure 4:**
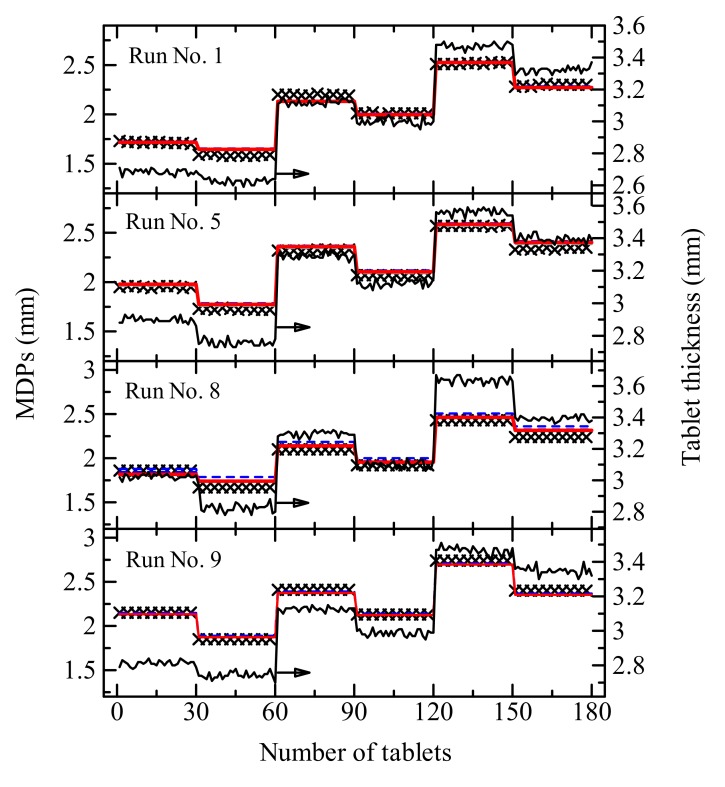
Actual setting (×) and predictive control of MDPs for the desired tablet weight and thickness by the NIR model (gray dashed line), physical attribute model (blue dashed line), and combination model (red solid line). The black solid line indicates actual thickness of tablets. The prediction was performed for batches of the four runs (No. 1, 5, 8, and 9) shown in Table 2. The prediction of No.9 data was performed as an external validation using the calibration model prepared excluding run No. 7 data. The desired values of tablet weight and thickness are shown in the figure.

The external validation of MDPs using the Form II excluded model resulted the same level of accuracy as the results of the internal validation. The predicted MDPs is shown in Figure 4. The tablet thickness is strongly dependent on MDPs; however, the thickness of the powder bed in the die became larger than the distance. This is because the plastic and elastic deformation and the rearrangement of particles occur as stress is applied to the powder bed, and the elastic recovery of the bed follows the decompression. Hence, the distance must be determined by controlling the viscoelasticity of the powder materials.

Based on the Heckel equation for the consolidation of powder particles, Ilkka et al. [29] studied the yield pressure of each material by the compression pressure profile and reported the dependence of the yield pressure on the nature of the material. Gustafsson et al. [30] predicted and analyzed the influence of hydroxypropyl methylcellulose (HPMC) characteristics on the compactibility by NIR and IR spectroscopy and suggested that the hydrogen bonding between the polymers might contribute to the compactibility. On the other hand, Gupta et al. [31] reported that moisture facilitated the compaction behavior of MCC. Quantifying the viscoelasticity and compaction property of each material is impossible; however, attributes such as the differences in hydrogen bonding, material contents, and moisture content can be determined from the NIR spectra. Hence, compared to the control model of FD, the chemical attributes significantly contributed to the model of MDPs, and the predicted values by the NIR model resulted in good agreement with the actual settings.

In order to implement the proposed control model for the continuous manufacturing system, several online and/or inline sensors are required for collecting not only the NIR spectra but also the physical attributes of particles size distribution, flowability, and density. In recent years, various NIR spectroscopic devices for online monitoring have become widely used [32]. Parsum (Malvern Panalytical, Worcestershire, UK) is an inline probe used to measure the particle size distribution and is also widely used for process monitoring [33]. The powder density may be predicted by the X-ray absorption coefficient [34]; however, the practical use of a density-monitoring sensor is challenging and difficult. As the one of the ideas to determine density, the volume and weight of the powder bed are measurable when the powder is blending with the lubricant before compression. The loose bulk density can be obtained as the density before lubricant blending, and the tapped density is equivalent to the density after the blending. When the effect of lubrication on the flowability of granules is negligible, the Hausner ratio and Carr index can be determined as the flowability indices. In case of direct compression, the lubrication improves the flowability of powders; thus, the direct compression process requires obtaining the flowability indices and density after the lubricant blending.

In the next stage, it is necessary to consider whether the control model can be applied as a complement to the existing built-in control or not. For example, the collecting positions of the chemical and physical attributes of the granules are important for optimal forward control; thus, the positions of in-line and/or on-line sensors must be specified using a rotary tableting machine. In addition, a command channel to the compression control system is required.

## 4. Conclusions

We established a robust feed-forward control model by predicting the process parameters based on the chemical and physical attributes of the granules. Because the chemical attributes of the granules can be determined by their NIR spectra, the NIR spectra were used to prepare a PLSR model to optimize the process parameters for a desired tablet weight and thickness. The parameters predicted by the model combining the NIR and physical attributes showed a reasonable agreement with the actual settings, and the error in the prediction was much lower than that in the previously reported model. There are several procedures involved in the preparation of tablets, such as powder blending, granulation, and drying. Among these procedures, it is believed that the feed-forward control of the tableting process is the most effective for realizing the final products of a targeted quality level. The chemical attributes of the granules made a smaller contribution to the optimization of FD but critically contributed to the determination of MDPs for the desired weight and thickness of a tablet. Although the feed-forward control of FD required additional data for more accurate prediction, we successfully demonstrated that the robust feed-forward control was feasible using PLSR with the combination of the NIR spectra and physical attributes of the granules. As a next step in the utilization of the proposed modelling method for the robust feed-forward control, it will be necessary to apply the method to a rotary tableting machine, since these machines are generally used for the large-scale production of tablets.

## Figures and Tables

**Table 1:** Test formulations of tablets.

Form.	LTD ^(1)^ (%)	AL ^(2)^ (%)	CS ^(3)^ (%)	MCC ^(4)^ (%)	HPC ^(5)^ (%)	MgSt ^(6)^ (%)
I	10	60	17.5	9.0	2.5	1.0
II	10	55	22.5	9.0	2.5	1.0
III	10	50	22.5	14	2.5	1.0

^(1)^ Loratadine; ^(2)^ Anhydrous lactose; ^(3)^ Corn starch; ^(4)^ Micro-crystalline cellulose; ^(5)^ Hydroxypropyl cellulose; ^(6)^ Magnesium stearate.

**Table 2:** High-shear wet granulation and drying process parameters.

Run No.	Form.^(1)^	Screw Speed ^(2)^ (rpm)	*V*_water_^(3)^ (mL)	Drying Time (h)	Dataset ^(4)^
1	I	1500	70	2	P
2	I	1500	80	2	C
3	I	1500	80	5	C
4	I	2000	70	2	C
5	I	2000	70	5	P
6	I	2000	80	2	C
7	II	2000	80	2	C(+,-)
8	III	2000	80	2	P
9	II	2000	80	5	P
10	III	2000	80	5	C
11	I	2500	70	2	C
12	I	2500	80	2	C
13	I	2500	80	5	C

^(1)^ Formulation of granules listed in Table 1; ^(2)^ Speed of side cross-screw; ^(3)^ Total volume of added water; ^(4)^ Dataset used for calibration (C) or prediction (P).

**Table 3:** Variations in the compression.

Tablet No.	Tablet Weight (mg)	Compression Pressure (MPa)
1	180	60
2	180	100
3	200	60
4	200	100
5	220	60
6	220	100

**Table 4:** Physical attributes of prepared granules.

Run No.	*ρ*_L_^(1)^ g/cm^3^	*ρ*_T_^(2)^ g/cm^3^	HR ^(3)^	CI ^(4)^ %	D10 μm	D50 μm	D90 μm
1	0.537	0.624	1.16	14.0	16	99	171
2	0.544	0.595	1.10	8.70	67	117	300
3	0.496	0.582	1.17	14.9	66	114	161
4	0.505	0.610	1.21	17.2	12	101	240
5	0.500	0.596	1.19	16.0	15	100	168
6	0.544	0.610	1.12	10.9	57	113	231
7	0.501	0.603	1.20	17.0	2.0	92	147
8	0.516	0.596	1.15	13.4	4.0	108	150
9	0.486	0.589	1.21	17.5	6.0	94	148
10	0.511	0.596	1.17	14.3	27	102	148
11	0.538	0.626	1.16	14.0	21	103	200
12	0.538	0.582	1.08	7.53	87	158	434
13	0.474	0.553	1.17	14.3	76	117	296
Ave.	0.515	0.597	1.16	13.8	37.8	109	215
S.D.	0.024	0.019	0.04	3.1	29.2	17	86

^(1)^ Loose density; ^(2)^ Tapped density; ^(3)^ Hasuner ratio; ^(4)^ Carr index.

**Table 5:** Calibration and cross-validation results of tableting process parameters based on the various input variables.

Target Parameter	Input Variables	LV ^(1)^	Calibration				Validation			
			Slope	Offset (mm)	RMSE ^(2)^ (mm)	*R* ^2^	Slope	Offset (mm)	RMSE ^(2)^ (mm)	*R* ^2^
FD	Physical	6	0.954	0.298	0.134	0.954	0.954	0.301	0.135	0.954
	NIR	6	0.968	0.209	0.112	0.968	0.967	0.211	0.113	0.968
	Combination ^(3)^	6	(+)0.969 (−)0.982	0.198 0.114	0.110 0.083	0.969 0.982	0.969 0.981	0.201 0.123	0.110 0.084	0.969 0.982
MDPs	Physical	5	0.969	0.0672	0.0489	0.969	0.969	0.0679	0.0491	0.969
	NIR	5	0.972	0.0598	0.0461	0.972	0.972	0.0603	0.0464	0.972
	Combination ^(3)^	5	(+)0.978 (−)0.980	0.0483 0.0442	0.0415 0.0400	0.978 0.980	0.978 0.979	0.0485 0.0456	0.0417 0.0403	0.977 0.979

^(1)^ Number of latent variables used; ^(2)^ Root mean square error; ^(3)^ The combination of physical and chemical attributes. The combination models were prepared with (+) and without (−) the data set of Run No. 7.

**Table 6:** Prediction error of FD and MDPs.

Target Param.	Input Variables	Slope	Offset (mm)	*R* ^2^	RMSE (mm)	Bias (mm)
FD	Physical	0.866	0.931	0.872	0.260	0.0634
	NIR	0.827	1.17	0.685	0.407	0.0515
	Combination ^(1)^	(+)0.914 (−)0.760	0.567 1.32	0.953 0.676	0.157 0.413	0.00839 0.228
MDPs	Physical	0.976	0.0663	0.980	0.0451	0.0163
	NIR	0.973	0.0775	0.973	0.0517	0.0200
	Combination ^(1)^	(+)0.963 (−)0.974	0.0905 0.102	0.983 0.926	0.0408 0.0862	0.0123 0.0481

^(1)^ The combination of physical and chemical attributes. The combination models were prepared with (+) and without (−) the data set of Run No. 7.
